# Supplementary material for: Case-area targeted interventions (CATI) for reactive dengue control: Modelling effectiveness of vector control and prophylactic drugs in Singapore
Source: PLoS Negl Trop Dis. 2021 Aug 11;15(8):e0009562. doi: 10.1371/journal.pntd.0009562 (PMC8357181; doi:10.1371/journal.pntd.0009562)
Supplement: S1 Fig — (DOCX) [file pntd.0009562.s001.docx]

## S1 Fig Plot of the entomological data over time

##
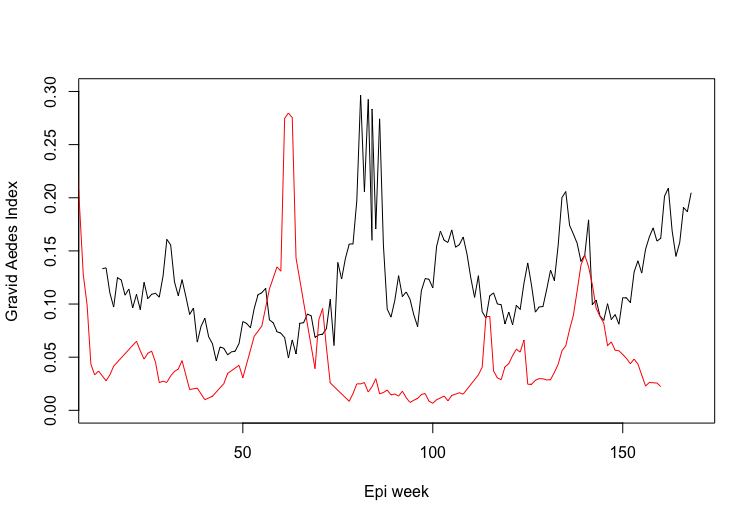


The black line shows the mean weekly value of the Gravid Aedes Index (GAI) with relative distribution of dengue cases over time superimposed in red. The entomological data can be viewed directly within the DENSpatial R package through the command data(mosq) or ?mosq
