# Supplementary material for: Case-area targeted interventions (CATI) for reactive dengue control: Modelling effectiveness of vector control and prophylactic drugs in Singapore
Source: PLoS Negl Trop Dis. 2021 Aug 11;15(8):e0009562. doi: 10.1371/journal.pntd.0009562 (PMC8357181; doi:10.1371/journal.pntd.0009562)
Supplement: S2 Fig — (DOCX) [file pntd.0009562.s002.docx]

## S2 Fig Distribution of human infectiousness


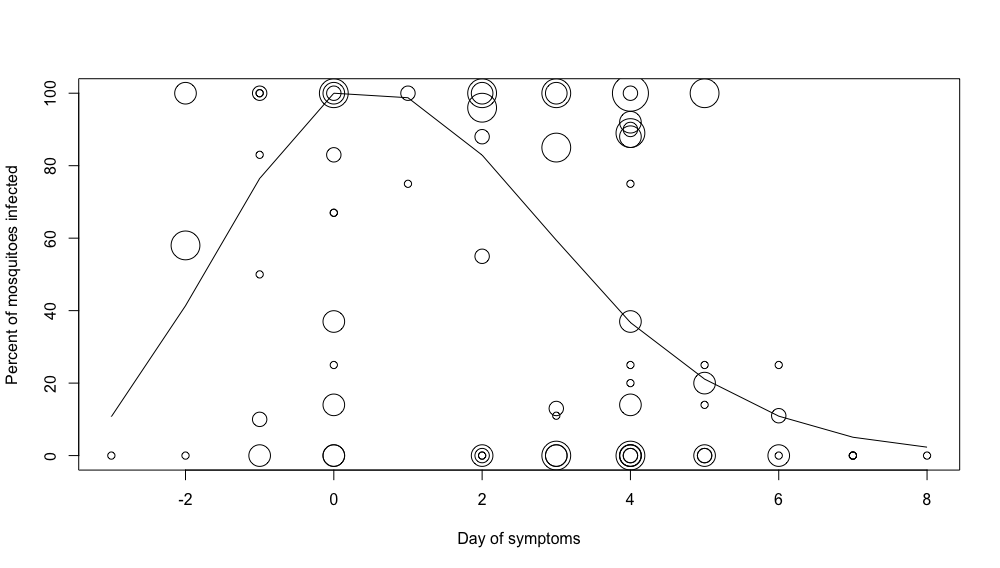


Binomial distribution fitted scaled to mosquito feeding data from [1] stratified by days since patient becomes symptomatic. Circle size is proportional to number patients sampled. Line of best fit was determined using the “fitdistrplus” package in R. Distribution is scaled to maximum values of 100% infectiousness. Following observations from Duong et al. [1] and Nguyen et al [2], infectiousness was predicted < 3 days and > 8 days becoming symptomatic.

References:

1. Duong V, Lambrechts L, Paul RE, Ly S, Lay RS, Long KC, et al. Asymptomatic humans transmit dengue virus to mosquitoes. Proc Natl Acad Sci U S A. 2015;112: 14688–93. doi:10.1073/pnas.1508114112

2. Nguyen MN, Duong THK, Trung VT, Nguyen THQ, Tran CNB, Long VT, et al. Host and viral features of human dengue cases shape the population of infected and infectious Aedes aegypti mosquitoes. Proc Natl Acad Sci U S A. 2013;110: 9072–7. doi:10.1073/pnas.1303395110
