# Supplementary material for: Case-area targeted interventions (CATI) for reactive dengue control: Modelling effectiveness of vector control and prophylactic drugs in Singapore
Source: PLoS Negl Trop Dis. 2021 Aug 11;15(8):e0009562. doi: 10.1371/journal.pntd.0009562 (PMC8357181; doi:10.1371/journal.pntd.0009562)
Supplement: S3 Fig — (DOCX) [file pntd.0009562.s003.docx]

## S3 Fig Map of estimated seroprevalence at baseline in Singapore


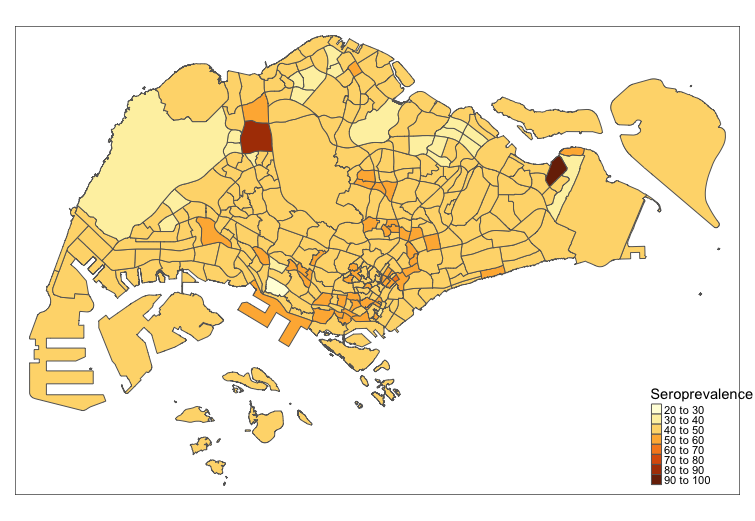


Map of predicted population-wide IgG seroprevalence in each planning area after taking into account differences in age distribution. The data can be further interrogated within the DENSpatial R package through the command data(sero) or ?sero. The base layer is the 2014 Master Plan subzone boundary for Singapore and contains information from Master Plan 2014 Subzone Boundary (Web) accessed on 20^th^ August 2020 from <https://data.gov.sg/dataset/master-plan-2014-subzone-boundary-web?resource_id=1c6b586b-61ca-45a9-b704-df4c9057fbd6> which is made available under the terms of the Singapore Open Data Licence version 1.0 <https://data.gov.sg/open-data-licence>
