# Supplementary material for: Case-area targeted interventions (CATI) for reactive dengue control: Modelling effectiveness of vector control and prophylactic drugs in Singapore
Source: PLoS Negl Trop Dis. 2021 Aug 11;15(8):e0009562. doi: 10.1371/journal.pntd.0009562 (PMC8357181; doi:10.1371/journal.pntd.0009562)
Supplement: S4 Fig — (DOCX) [file pntd.0009562.s004.docx]

## S4 Fig Model fit to each case cluster

Shows the fit of the DENSpatial model to different case clusters (rows) assuming different human movement models (model 1 = exponential, model 2 = gravity, model 3 = radiation). Different coloured histograms describe the distribution of distance values (distance function between model predictions and observed data, d) over the three successive rounds of Sequential Monte Carlo (SMC, first round red, last round blue). Text summary metrics in the top right of each plot give percentage improvements in reduction of the distance function between rounds 1 and 3 of SMC, including the breakdown by spatial and temporal components.
