# Supplementary material for: Case-area targeted interventions (CATI) for reactive dengue control: Modelling effectiveness of vector control and prophylactic drugs in Singapore
Source: PLoS Negl Trop Dis. 2021 Aug 11;15(8):e0009562. doi: 10.1371/journal.pntd.0009562 (PMC8357181; doi:10.1371/journal.pntd.0009562)
Supplement: S1 Text — (DOCX) [file pntd.0009562.s005.docx]

## S1 Text Population map generation

Singapore building-level data was extracted from OpenStreetMap ([www.openstreetmap.org](http://www.openstreetmap.org)) on 20^th^ August 2020. This database contains user digitised polygons for a variety of building types and land uses. To narrow these categories down to places where humans spend significant time indoors (given *Ae. aegypti* preferentially bites indoors) we excluded a number of land categories from this dataset. The full list of exclusions is given in table A

Table A

| Land use exclusions | Land category exclusions |
| --- | --- |
| Allotmants | Aeroway |
| Aquaculture | Boundary |
| Basin | Leisure |
| Brownfield | Military |
| Cemetary | place |
| Churchyard | Natural |
| Disused | Man_made |
| Container_terminal | barrier |
| Depot |  |
| Farmland |  |
| Farmyard |  |
| Forest |  |
| Grass |  |
| Greenfield |  |
| Greenhouse_horticulture |  |
| Landfill |  |
| Meadow |  |
| military |  |
| Orchard |  |
| Plant_nursary |  |
| Proposed |  |
| Quarry |  |
| Railway |  |
| Reservoir |  |
| Scrub |  |
| Village_green |  |
| Winter_sports |  |
| industrial |  |
| Changi airport construction zone |  |

As a final filter any polygons larger than 500m x 500m were excluded which further identified large construction zones and country parks not identified by the above exclusion categories.

Next the building levels information was extracted from each polygon object and summed over pixels at a resolution of 0.0008333 decimal degrees (~ 100m). Where building level information was not available we assumed structures had only a single floor. Population counts at the planning area level from 2020 department of statistics projections ^1^ were then divided among populated pixels proportional to the aggregate number of floors per pixel. The final derived population map is shown in Fig A and can be further examined within the DENSpatial R package through the command data(sgpop) or ?sgpop.

Fig A: Derived map of population (residents per pixel) in SingaporeThe base layer is a rasterised version of the 2014 Master Plan subzone boundary for Singapore and contains information from Master Plan 2014 Subzone Boundary (Web) accessed on 20^th^ August 2020 from <https://data.gov.sg/dataset/master-plan-2014-subzone-boundary-web?resource_id=1c6b586b-61ca-45a9-b704-df4c9057fbd6> which is made available under the terms of the Singapore Open Data Licence version 1.0 <https://data.gov.sg/open-data-licence>

References

1. Department of Statistics Singapore. *Population Trends, 2020*. Singapore; 2020. doi:2591-8028
