# Supplementary material for: Case-area targeted interventions (CATI) for reactive dengue control: Modelling effectiveness of vector control and prophylactic drugs in Singapore
Source: PLoS Negl Trop Dis. 2021 Aug 11;15(8):e0009562. doi: 10.1371/journal.pntd.0009562 (PMC8357181; doi:10.1371/journal.pntd.0009562)
Supplement: S2 Text — (DOCX) [file pntd.0009562.s006.docx]

## S2 Text Equations for human movement models

Human movement between patch $i$ and patch $j$ ($T_{i\to j}$) can is represented as one of three different human movement models:

*Exponential:*

$$T_{i\to j}=\frac{1}{e^{kd_{i\to j}}}$$

*Gravity:*

$$T_{i\to j}=\frac{\left( p_{i}+p_{j} \right)}{kd_{i\to j}}$$

*Radiation:*

$$T_{i\to j}= \frac{p_{i}p_{j}}{{(p}_{i}+{ks}_{ij})(p_{i}+p_{j}+ks_{ij})}$$

where:

$d_{i\to j}$ = Euclidean distance between patch $i$ and patch $j$

$p_{i}$ = population in patch $i$

$s_{ij}$= total population living within the distance$d_{i\to j}$ of patch $i$

$k$ = distance scaling parameter fit using the case data

These fluxes were interpreted as the proportion of time in an individual’s day that they spent in each patch. Fluxes for each patch were normalised ($\sum_{i}^{j} T_{ij}=1$) and multiplied by the number of individuals resident in patch $i$ to give the total time allocation of all individuals resident in patch $i$.
