# Supplementary material for: Case-area targeted interventions (CATI) for reactive dengue control: Modelling effectiveness of vector control and prophylactic drugs in Singapore
Source: PLoS Negl Trop Dis. 2021 Aug 11;15(8):e0009562. doi: 10.1371/journal.pntd.0009562 (PMC8357181; doi:10.1371/journal.pntd.0009562)
Supplement: S1 Table — (DOCX) [file pntd.0009562.s007.docx]

## S1 Table Sensitivity of clustering algorithm to choice of linkage break threshold

Percentage similarity in classification of dengue clusters with clustering algorithms with different linkage threshold values. Table shows % of records with the same classification with the default threshold value (links in the 95^th^ percentile = within cluster links)

| Human movement model | Exponential (%) | Gravity | Radiation |
| --- | --- | --- | --- |
| Links in the 90^th^ percentile | 100 | 100 | 100 |
| Links in the 99^th^ percentile | 100 | 100 | 90 |
