# Supplementary material for: Case-area targeted interventions (CATI) for reactive dengue control: Modelling effectiveness of vector control and prophylactic drugs in Singapore
Source: PLoS Negl Trop Dis. 2021 Aug 11;15(8):e0009562. doi: 10.1371/journal.pntd.0009562 (PMC8357181; doi:10.1371/journal.pntd.0009562)
Supplement: Attachment — Submitted filename: 01_Response_to_reviewers.docx [file pntd.0009562.s009.docx]

Faculty of Epidemiology and Public Health

London School of Hygiene and Tropical Medicine

Keppel Street, London, WC1E 7HT, U.K.

E-mail: oliver.brady@lshtm.ac.uk

Dr. Hannah Clapham and Dr. Nigel Beebe

*PLoS NTDs*

28^th^ April 2020

**Covering letter for resubmission of: “Case-area targeted interventions (CATI) for reactive dengue control: modelling effectiveness of vector control and prophylactic drugs in Singapore” (PNTD-D-20-02174) - [EMID:f86d61e2ee9cba7f]**

Dear Drs. Clapham and Beebe

Thank you for giving us the opportunity to revise out submission of our dengue case area targeted interventions modelling paper. We found the comments of the reviewers highly valuable for clarifying the scope and details of our work. We have detailed our responses and additions in a point-by-point summary found below.

Please let us know if further revisions or clarifications are required.

Yours sincerely on behalf of all co-authors

Oliver J Brady, Associate Professor

LSHTM, London, UK

Reviewer #1: I have several questions regarding the validity and applicability of the analysis.

1) GAI index used for mosquito abundance, how is this sampled? Can it affect results if unequal sampling has been carried out across Singapore?

We agree with the reviewer that the representativeness of the GAI sampling is important and have now included a reference to a paper describing the sampling design in detail, including how it ensured representative sampling of Singapore across its 34 sites (Ong et al. 2020) ^1^.

2) Patches, is 1km x 1km appropriate? A lot of area based heterogeneity, which may not be represented within patch dynamics if grids are just defined by "squares". Furthermore, many barriers exist within Singapore, some porous, others not so porous. These will significantly affect spatial mosquito distributions where 1km will not capture small scale sudden outbreaks - by the time an area of 1km is notifying a case surge, it is a fire fighting regional effort to control mosquito populations and transmission and therefore outbreaks are not really averted. Microscale treatment around houses would not avert an outbreak.

We agree with the reviewer that dengue transmission is highly heterogeneous, particularly over shorter timescales and in smaller outbreaks, placing a limit on the inferences that can be made about very small-scale transmission patterns from this analysis.

In this paper we focus on the use of CATI strategies for larger outbreak control across larger areas (50-1000m radii). As mentioned in the introduction: “there is evidence to suggest that dengue infection is highly spatiotemporally clustered either in the household or within the local area (up to 1km) ^2–5^”. This suggests that, at least in major outbreaks in many settings, transmission is fairly well mixed at the scale of 0-1000m. This guided our choice of a 1km x 1km patch size. We should clarify that while the model is implemented on a 1km x 1km grid network we are still able to model the effects of finer-scale transmission and intervention policies, such as the 50-100m radii in figure 7. We are able to do this because, based on the above evidence ^2–5^, assume individuals homogeneous mix at finer scales and that features of the environment (mosquito population size and human immunity) are broadly similar at this scale, which we believe to be an acceptable approximation for assessing the effects of CATI interventions during large outbreaks.

We clearly acknowledge this limitation and its consequences in the discussion section:

“Our model likely underrepresents geographic heterogeneity in mosquito-human contact, particularly in Singapore where small-scale construction sites are known to be significant contributors to overall transmission ^6^. Higher geographic heterogeneity would make individual hotspots harder to control, but potentially more susceptible to focal targeting if they can be successfully identified.”

This clarification and additional detail of our assumptions has now been added to the first part of the Methods section:

“This population raster was then aggregated to 1km x 1km pixel level then rounded to the nearest integer for all subsequent model-based analyses. This 1km x 1km patch size choice was based on previous work that suggests dengue transmission in large outbreaks tends to be highly spatially clustered up to 1km) ^2–5^. For clarity, this does while the model is implemented on a 1km x 1km patch scale, the model is still able to track fienr-scale transmission events and intervention policies by assuming individuals homogeneous mix and that features of the environment (mosquito population size and human immunity) are broadly similar at sub 1km x 1km scales. We believe to be an acceptable approximation for assessing the effects of CATI interventions during large outbreaks.”

3) Calibration of model to all years? Or only within epidemic season? How was model fit overall on epidemic trajectory? Does it replicate observed seasons over time? Measure of correlation between fit and observation (R^2) possible?

As described in the methods (section “Model fitting”), the Results (section “Model fit results”) and in figures 3 and 4, the model is fit to the three primary clusters over the period May 2013 – June 2016, which include parts of the epidemic and inter-epidemic season.

The principal characteristics we want the model to fit in the data is the relative distribution of cases over space and time, hence why they are prioritised in the model fitting procedure over measures of epidemic trajectory or seasonality. Including additional terms in the model fit would compromise the fit of the model to the distribution of cases over space and time and hence give inaccurate estimates of the effect of CATI strategies which was our primary goal.

The fit of the model is presented in Figure 5 with summary statistics given in the relevant parts of the results (section “Model fit results”). Because our primary fit metric is the relative distribution of cases over space and time there is no direct pairing between observation and prediction that would be necessary to generate a meaningful R^2 metric. Instead, what we are trying to replicate with the model fit is approximately equal distribution of transmission events at different distance intervals which is shown in figure 5. We also quote key statistics in the relevant parts of the results section e.g. “The models estimate comparable within patch transmission (77%, 70% and 61% for clusters 1-3)”

4) How will between serotype interactions affect the results? Singapore is hyper-endemic. Serotype switching is well documented to pre-empt looming epidemics.

In the “model structure” section of the methods we clarify why we chose the approximation of a single serotype model and justify this assumption:

“Despite the co-circulation of multiple serotypes in Singapore both past and present, the outbreaks between 2013 and 2015 were predominantly due to DENV1 (70-90% of reported cases), as were the majority of cases leading up to the period of interest with the exception of a brief period of DENV2 circulation 2007-2012 ^7,8^. With a focus on only modelling the dynamics of the outbreaks in question we assume only a single serotype model with sterilising infection. Given the existence of temporary cross serotype immunity following recent infection with any one dengue type and the predominance of one type, we would expect similar results from a multi-serotype model over the short timeframe over which our model is focussed.”

However, we do agree and acknowledge that serotype switching may be required to explain some of the more complex finer scale spatial patterns and suggest it could be the focus of future work in the discussion:

“This may require developing a multi-serotype model to reflect co-circulation of multiple DENV serotypes and the complex pattern of human immunity that they impose in settings like Singapore.”

5) The differences in movement types (gravity, radiation, exponential) has been well studied elsewhere. Of great usefulness would be to use a fitted movement distribution for mosquitoes rather than theoretical ones, in a smaller scale study area/areas where mosquito gravitrap data has been collected. This would be much more convincing of CATI's usefulness.

We do agree that spatial heterogeneity in mosquito abundance and mosquito-human contact rate are important.

Consistent with previous studies ^2,9,10^ at the scale the model is implemented (1km x 1km patches) we assume that spread of dengue virus transmission between patches is primarily facilitated by movement of humans rather than movement of mosquitoes and we represent these movements of humans using gravity, radiation and exponential movement models. Previous mark release recapture studies of *Ae. aegypti* in Singapore have suggested very limited (<50 m) flight ranges if they leave the premises at all ^11^ which guided our decision to not include mosquito movement between patches in the model.

In the current work we estimate the mean mosquito-human contact rate and its degree of correlation with previous human exposure to dengue virus. This allows us to represent basic heterogeneities in the mosquito population across Singapore. Ideally, we would have entomological data that could represent this heterogeneity, however the GAI study was not powered to detect differences across space as well as time. This limitation is acknowledged in the discussion:

“Over the time period of our data entomological surveys were only available from a small number of sentinel sites that were sufficient to measure variation in mosquito abundance over time, but not space at a sufficiently high resolution. If entomological data were available at higher spatial resolution the model may have been able to better capture the observed spatial heterogeneity in dengue cases ^12^”

6) In reading CATI: [https://journals.plos.org/plosmedicine/article?id=10.1371/journal.pmed.1002509](https://protect-eu.mimecast.com/s/CNZaCwr14SvkQpGuQ18bT?domain=journals.plos.org), Singapore has referenced "CATI" simply as vector control and has been carrying this out since vector control practices began. The use of a prophylatic drug (which has been shown to be applicable to cholera) has a myriad of issues for dengue in terms of lifespan of the drug and whether such a drug will be effective in such a scheme in terms of deployment - if a prophylatic is shown to be successful, population wide implementation or the arrival of a new serotype would be much more advantageous as a signal of an upcoming epidemic.

We agree with the reviewer that the use of a prophylactic drug for dengue has many issues, all of which will require further detailed research. In this analysis we aim to answer just a small part of this problem in answering the question: if a suitable prophylactic drug could be developed, would it be suitable to use it in a reactive CATI strategy? We assume a hypothetical drug with lifespan of 30 days. We agree that more research needs to be done to test how the effectiveness of CATI with prophylactic drugs changes with different drug lifespans, but think such work will be most relevant when such parameters are known from Phase 1/2 trials. We have added this important point to the relevant part of the discussion:

“Following Phase 1 and 2 trials, more detailed modelling studies will be necessary to explore how the effectiveness of CATI strategies with prophylactic drugs changes with the specific characteristics (e.g. duration of efficacy) of each drug.”

We also agree that CATI should be compared to alternative strategies and in particular population-wide implementation. However, given the focus of model development and CATI strategies we believe this is outside the scope of the current paper and should be addressed in future research.

While the existing vector control response in Singapore may include some case-reactive elements, our analysis is still useful for understanding how the scale (radius) and timeliness affects the effectiveness of such practices.

Reviewer #2: The model design makes it clear how CATI effectiveness will be measured, and all data is made readily available. There are some gaps not addressed when comparing the model structure to the DENV transmission pattern that are explained in the notes attached. In essence, not allowing individuals to become susceptible again may obscure the relationship between DENV prevalence and dengue burden. Given the significant baseline seroprevalence (46%), individuals appear likely to be infected with DENV more than once regardless of number of circulating serotypes.

We agree with the reviewer that infections occurring within Singapore within this time period are likely to be a mixture of primary, secondary, tertiary and quaternary infections. In our model we use the term “Susceptible” to indicate susceptibility to the type of dengue co-circulating at the time of observation. As such this definition is inclusive of individuals who have been previously infected with dengue at some point in their lifetime (and would thus test seropositive in an IgG serosurvey prior to infection). This may mean that the 48.6% seropositivity over estimates the proportion immune in our model initial conditions. However, we believe this overestimation is likely to be minimal because, as mentioned in the Initial conditions section of the Methods: “Dengue virus serotype 1 has predominated (accounting for 70-90% clinical cases) since Singapore began DENV serotype surveillance with the exception of the years of 2007-2012 ^8,13^”. Even the effects of the brief period of non-DENV-1 predominance in 2007-2012 is likely to be minimal due to the effects of temporary cross immunity (as mentioned in the Model structure section of the methods where we justify our choice of single serotype model).

As for why our model does not structurally represent transition of individuals back to the susceptible compartment, this is mainly due to the limited time window of our analysis. The analysis covers May 2013 – June 2016 with the vast majority of infections within each cluster occurring within a single year (Figure 3). Evidence from cohort studies^14^ and large observational studies^15^ suggest that cross-serotype temporary cross immunity lasts between 1 and 3 years, meaning that even if some of the outbreaks in our fitting dataset were caused by different serotypes, is unlikely that a given individual would be infected more than once over the period of this study.

Beyond this, when considering whether CATI strategies are effective, it may be nice to expand on how prevention of onward transmissions relates to actual reduction in disease burden. This is somewhat dependent on the ability to number infections, but rates of disease based on infection number are described here. [https://journals.plos.org/plosmedicine/article?id=10.1371/journal.pmed.1002181](https://protect-eu.mimecast.com/s/CBM8Cx1g4cLD0O1uA4OBY?domain=journals.plos.org)

We agree with the reviewer that quantifying the benefits of any intervention in terms of disease burden reduction, in addition to infection reduction, is important. In this paper we primarily focus on the question of whether CATI strategies can prevent outbreaks over seasonal (up to 1 year) time horizons. This shaped our decision to approximate transmission with a single-serotype model with relatively short-term predictions and means we are unable to separate infections by infection order (i.e. primary, secondary, etc) and attach severity weights based on Flasche et al. as suggested. We do give estimates of disease burden reduction because our measure of effectiveness is based on the reduction in symptomatic cases (e.g. Fig. 6), but these are estimated by a single parameter that dictates the probability that an infection is symptomatic, correctly diagnosed and reported and does not distinguish severe and non-severe disease.

The question of whether these averted infections are truly averted or just delayed and the impact this may have on the probability of the individual to experience further, possibly severe, infections within their lifetime is an important one, but would require substantial modification of our model or perhaps an entirely different approach to capture sequenced infection dynamics and long-term population changes.

We have added the following section to the discussion to highlight the importance of this in subsequent work:

“Developing multiple infection models with realistic population dynamics will also be important for projecting the longer-term reductions in disease burden attributable to CATI. This is important as it remains unknown whether the infections CATI averts are truly averted or just delayed until later in an individual’s lifetime.”

From the model fit on, results match what is described in the methods and background section. The section starting on line 403 is confusing as is. It seems that 100% effectivity is assumed for both vector and prophylactic solutions, an unrealistic standard in dengue prevention. If this is instead intended to describe model behavior or assumptions, the heading on line 400 should be changed to reflect this.

We thank the reviewer for pointing out the discrepancy between this first theoretical model (where we assume 100% effective coverage) and the latter model simulation parts of the results section (where we assume 90% efficacy and 90% coverage to give an effective coverage of 81%). In the second line of the results we have clarified this:

“This simple model assumed that vector control and prophylactic drugs had 100% efficacy and that delays in their application was the only factor that limited effectiveness.”

Figures are easy to understand and work well in context. Figure 8’s y-axis should be changed to say “Relative number of infections”

Thanks for spotting this typo, it has now been corrected.

Results are contextualized well, and limitations of model are appropriately considered. I would suggest that locations that are fit for CATI be explicitly mentioned, and put in contrast with locations where CATI is not appropriate. I noted this in methods, but I believe that rates of symptomatic cases by serial infection and the CYD-TDV vaccine should be mentioned here as well.

We agree that it is a good idea to mention alternative strategies where we predict CATI is unlikely to be effective, including use of the CYD-TDV vaccine. As we do not model the effects of any alternative interventions we have included this as part of the discussion with the following new sentence:

“This also highlights the need for targeting preventative interventions such as environmental clean-up campaigns and vaccines (e.g. CDY-TDV) in high density, high transmission areas which may then allow CATI to contain outbreaks where it previously could not.”

As the data we fit our model to only covers a brief time period of dengue activity in Singapore we are hesitant to map locations of high and low predicted CATI effectiveness as we are unsure how well such maps would generalise over time. Instead, what we do is try to identify the principal features of high and low effectiveness environments that may give generalisable insights into where CATI may work better or worse (figure 6B).

This paper does a good job at describing CATI and its appropriate role in dengue control. Its model supports the conclusions drawn and its limitation and sensitivities are described well.

We thank the reviewer for this encouraging comment.

Specific comments:

Line 84: change “current outbreaks” to “current dengue outbreaks”

changed

Line 101: Suggested citations

- <https://journals.plos.org/ploscompbiol/article?id=10.1371/journal.pcbi.1006710>

Have included

Line 104: CYD-TDV is currently licensed in Singapore, although there are strict warnings. <https://www.hsa.gov.sg/docs/default-source/hprg-vcb/pem-pmg-pac/dengvaxia-pmg-v2_30082018.pdf>. Singapore is also home to several studies where CYD-TDV behavior is measured: <https://www-tandfonline-com.offcampus.lib.washington.edu/doi/full/10.1080/21645515.2019.1578595>

Have changed to “while no drug is currently used”

Line 120: Suggested citations

- <https://www.sciencedirect.com/science/article/pii/S0264410X18303281>
- Rodríguez-Barraquer I, Salje H, Cummings DA. Dengue pre-vaccination screening and positive predictive values. Lancet Infect Dis. 2019 Feb;19(2):132-134. doi: 10.1016/S1473-3099(18)30799-0. Epub 2019 Jan 30. PMID: 30712834.
- <https://journals.plos.org/plosntds/article?id=10.1371/journal.pntd.0000545>
- <https://www.sciencedirect.com/science/article/pii/S0001706X09003556>
- <https://journals.plos.org/plosntds/article?id=10.1371/journal.pntd.0000670>
- <https://journals.plos.org/plosntds/article?id=10.1371/journal.pntd.0005317&rev=2>

Line 120: “Two theoretical modelling studies of CATI have been undertaken for dengue [14,15]…” relates to previous studies that assessed the effectiveness of spatially targeted interventions rather than studies that assessed the spatial concentration of transmission. We therefore believe that the above studies are not suitable references for this section. We have clarified this focused scope by changing the sentence to read:

“Two theoretical modelling studies of spatially reactively targeted interventions (CATI) for outbreak control have been undertaken for dengue”

Lines 132-135: Considers targeting CYD-TDV vaccination in high burden areas where DENV shows spatial heterogeneity.

- <https://www.sciencedirect.com/science/article/pii/S2468042720300531>

Similar to the above comment we draw the distinction between studies that aim to characterise spatial heterogeneity in transmission as a basis for targeting interventions, of which there are many, and modelling studies that test the effectiveness of real-time spatially reactive intervention targeting, of which there are few.

Line 189: change “to multiple the” to “to multiply the”

Done

Line 202: This needs to be moved up when talking about the model structure as it dictates human movement through compartments

Line 202 (Line beginning “Despite the co-circulation of multiple serotypes in Singapore…”) is te beginning of our justification of why we choose to approximate transmission with a single serotype model and is already within the “Model structure” section of the methods. Paragraphs above this also describe the compartments of the model and how individuals move between compartments.

Line 213: This line in like of line 173 is confusing. Line 173 suggests that humans don’t have an exposed compartment.

The legend to figure 1 has been clarified to indicate that only the mosquito population has an exposed compartment as this is addressed in humans by having time-varying infectiousness within the “Infected” compartment

Line 318: prophylactic against disease or infection? Generally I think these assumptions are too strong based on the current state of Dengue vaccine development.

Line 318-9 states “In the absence of an internationally recognised target product profile for dengue prophylactics, here we consider a drug with 90% protective efficacy against infection…”. We believe that the short-term efficacy of a continually applied drug is likely to be higher than the long-term protection afforded by vaccination. Similar relative effectiveness between drugs and vaccines has been observed for malaria.

Line 329: Maybe put assumed for parameters that weren’t based in evidence.

Done

Line 406: Generally this isn’t acknowledging the different rates of disease by numbered infection. This is particularly important considering that 46% of the population has at least one prior infection.

See response to point 1 of Reviewer #2

Line 412: change to “We find that even short delays in detection or response”, confusing as is.

Done

Paragraph starting on 412: Prior to this, is there anywhere that states that vector control is assumed to be 100% effective? I was surprised to see that in Fig. 2B, and don’t know if that is plausible

Line 421: Again, make clear the assumption is that the prophylactic drug is 100% effective at preventing onward transmission

For both comments, see response to point 3 of Reviewer #2

Line 610: can you provide examples of these types of locations?

see response to point 5 of Reviewer #2

**Bibliography**

1.	Ong J, Chong C-S, Yap G, et al. Gravitrap deployment for adult Aedes aegypti surveillance and its impact on dengue cases. Aldridge RL, ed. *PLoS Negl Trop Dis*. 2020;14(8):e0008528. doi:10.1371/journal.pntd.0008528

2.	Anders KL, Nga LH, Thuy NT Van, et al. Households as Foci for Dengue Transmission in Highly Urban Vietnam. *PLoS Negl Trop Dis*. 2015;9(2):e0003528. doi:10.1371/journal.pntd.0003528

3.	Liebman KA, Stoddard ST, Morrison AC, et al. Spatial Dimensions of Dengue Virus Transmission across Interepidemic and Epidemic Periods in Iquitos, Peru (1999–2003). *PLoS Negl Trop Dis*. 2012;6(2):e1472. doi:10.1371/journal.pntd.0001472

4.	Salje H, Lessler J, Maljkovic Berry I, et al. Dengue diversity across spatial and temporal scales: Local structure and the effect of host population size. *Science*. 2017;355(6331):1302-1306. doi:10.1126/science.aaj9384

5.	Guzzetta G, Marques-Toledo CA, Rosà R, Teixeira M, Merler S. Quantifying the spatial spread of dengue in a non-endemic Brazilian metropolis via transmission chain reconstruction. *Nat Commun*. 2018;9(1):2837. doi:10.1038/s41467-018-05230-4

6.	Liang S, Hapuarachchi HC, Rajarethinam J, et al. Construction sites as an important driver of dengue transmission: implications for disease control. *BMC Infect Dis*. 2018;18(1):382. doi:10.1186/s12879-018-3311-6

7.	Amin FM, Kheong CC, Chem Y, et al. 2013 Dengue Outbreaks in Singapore and Malaysia Caused by Different Viral Strains. *Am J Trop Med Hyg*. 2015;92(6):1150-1155. doi:10.4269/ajtmh.14-0588

8.	Lee KS, Lai YL, Lo S, et al. Dengue virus surveillance for early warning, Singapore. *Emerg Infect Dis*. 2010;16(5):847-849. doi:10.3201/eid1605.091006

9.	Stoddard ST, Forshey BM, Morrison AC, et al. House-to-house human movement drives dengue virus transmission. *Proc Natl Acad Sci U S A*. 2013;110(3):994-999. doi:10.1073/pnas.1213349110

10.	Perkins TA, Reiner RC, España G, et al. An agent-based model of dengue virus transmission shows how uncertainty about breakthrough infections influences vaccination impact projections. *PLoS Comput Biol*. 2019;15(3):e1006710. doi:10.1371/journal.pcbi.1006710

11.	Liew C, Curtis CF. Horizontal and vertical dispersal of dengue vector mosquitoes, Aedes aegypti and Aedes albopictus, in Singapore. *Med Vet Entomol*. 2004;18(4):351-360. doi:10.1111/j.0269-283X.2004.00517.x

12.	Ong J, Liu X, Rajarethinam J, et al. Mapping dengue risk in Singapore using Random Forest. *PLoS Negl Trop Dis*. 2018;12(6):e0006587. doi:10.1371/journal.pntd.0006587

13.	Hapuarachchi HC, Koo C, Rajarethinam J, et al. Epidemic resurgence of dengue fever in Singapore in 2013-2014: A virological and entomological perspective. *BMC Infect Dis*. 2016;16(1):300. doi:10.1186/s12879-016-1606-z

14.	Reiner RC, Stoddard ST, Forshey BM, et al. Time-varying, serotype-specific force of infection of dengue virus. *Proc Natl Acad Sci*. 2014;111(26):E2694-702. doi:10.1073/pnas.1314933111

15.	Reich NG, Shrestha S, King AA, et al. Interactions between serotypes of dengue highlight epidemiological impact of cross-immunity. *J R Soc Interface*. 2013;10(86). doi:10.1098/rsif.2013.0414
