# Supplementary material for: Case-area targeted interventions (CATI) for reactive dengue control: Modelling effectiveness of vector control and prophylactic drugs in Singapore
Source: PLoS Negl Trop Dis. 2021 Aug 11;15(8):e0009562. doi: 10.1371/journal.pntd.0009562 (PMC8357181; doi:10.1371/journal.pntd.0009562)
Supplement: Attachment — Submitted filename: reviewer comments.docx [file pntd.0009562.s008.docx]

**General comments**

Line 84: change “current outbreaks” to “current dengue outbreaks”

Line 101: Suggested citations

- <https://journals.plos.org/ploscompbiol/article?id=10.1371/journal.pcbi.1006710>

Line 104: CYD-TDV is currently licensed in Singapore, although there are strict warnings. <https://www.hsa.gov.sg/docs/default-source/hprg-vcb/pem-pmg-pac/dengvaxia-pmg-v2_30082018.pdf>. Singapore is also home to several studies where CYD-TDV behavior is measured: <https://www-tandfonline-com.offcampus.lib.washington.edu/doi/full/10.1080/21645515.2019.1578595>

Line 120: Suggested citations

- <https://www.sciencedirect.com/science/article/pii/S0264410X18303281>
- Rodríguez-Barraquer I, Salje H, Cummings DA. Dengue pre-vaccination screening and positive predictive values. Lancet Infect Dis. 2019 Feb;19(2):132-134. doi: 10.1016/S1473-3099(18)30799-0. Epub 2019 Jan 30. PMID: 30712834.
- <https://journals.plos.org/plosntds/article?id=10.1371/journal.pntd.0000545>
- <https://www.sciencedirect.com/science/article/pii/S0001706X09003556>
- <https://journals.plos.org/plosntds/article?id=10.1371/journal.pntd.0000670>
- <https://journals.plos.org/plosntds/article?id=10.1371/journal.pntd.0005317&rev=2>

Lines 132-135: Considers targeting CYD-TDV vaccination in high burden areas where DENV shows spatial heterogeneity.

- <https://www.sciencedirect.com/science/article/pii/S2468042720300531>

Line 189: change “to multiple the” to “to multiply the”

Line 202: This needs to be moved up when talking about the model structure as it dictates human movement through compartments

Line 213: This line in like of line 173 is confusing. Line 173 suggests that humans don’t have an exposed compartment.

Line 318: prophylactic against disease or infection? Generally I think these assumptions are too strong based on the current state of Dengue vaccine development.

Line 329: Maybe put assumed for parameters that weren’t based in evidence.

Line 406: Generally this isn’t acknowledging the different rates of disease by numbered infection. This is particularly important considering that 46% of the population has at least one prior infection.

Line 412: change to “We find that even short delays in detection or response”, confusing as is.

Paragraph starting on 412: Prior to this, is there anywhere that states that vector control is assumed to be 100% effective? I was surprised to see that in Fig. 2B, and don’t know if that is plausible

Line 421: Again, make clear the assumption is that the prophylactic drug is 100% effective at preventing onward transmission

Line 610: can you provide examples of these types of locations?
